# Tolerance to mild salinity stress in japonica rice: A genome-wide association mapping study highlights calcium signaling and metabolism genes

**DOI:** 10.1371/journal.pone.0190964

**Published:** 2018-01-17

**Authors:** Julien Frouin, Antoine Languillaume, Justine Mas, Delphine Mieulet, Arnaud Boisnard, Axel Labeyrie, Mathilde Bettembourg, Charlotte Bureau, Eve Lorenzini, Muriel Portefaix, Patricia Turquay, Aurore Vernet, Christophe Périn, Nourollah Ahmadi, Brigitte Courtois

**Affiliations:** 1 Centre de coopération internationale en recherche agronomique pour le développement, Unité mixte de recherche Amélioration génétique et adaptation des plantes méditerranéennes et tropicales, Montpellier, France; 2 Centre Français du Riz, Arles, France; 3 Institut National de la Recherche Agronomique, Unité mixte de recherche Amélioration génétique et adaptation des plantes méditerranéennes et tropicales, Montpellier, France; Louisiana State University, UNITED STATES

## Abstract

Salinity tolerance is an important quality for European rice grown in river deltas. We evaluated the salinity tolerance of a panel of 235 temperate japonica rice accessions genotyped with 30,000 SNP markers. The panel was exposed to mild salt stress (50 mM NaCl; conductivity of 6 dS m^-1^) at the seedling stage. Eight different root and shoot growth parameters were measured for both the control and stressed treatments. The Na^+^ and K^+^ mass fractions of the stressed plants were measured using atomic absorption spectroscopy. The salt treatment affected plant growth, particularly the shoot parameters. The panel showed a wide range of Na^+^/K^+^ ratio and the temperate accessions were distributed over an increasing axis, from the most resistant to the most susceptible checks. We conducted a genome-wide association study on indices of stress response and ion mass fractions in the leaves using a classical mixed model controlling structure and kinship. A total of 27 QTLs validated by sub-sampling were identified. For indices of stress responses, we also used another model that focused on marker × treatment interactions and detected 50 QTLs, three of which were also identified using the classical method. We compared the positions of the significant QTLs to those of approximately 300 genes that play a role in rice salt tolerance. The positions of several QTLs were close to those of genes involved in calcium signaling and metabolism, while other QTLs were close to those of kinases. These results reveal the salinity tolerance of accessions with a temperate japonica background. Although the detected QTLs must be confirmed by other approaches, the number of associations linked to candidate genes involved in calcium-mediated ion homeostasis highlights pathways to explore in priority to understand the salinity tolerance of temperate rice.

## Introduction

In Europe, rice is grown on approximately 437,000 ha [1]. Italy (220,000 ha) and Spain (110,000 ha) are the main producers, while Greece, Portugal and France are smaller producers (each under 30,000 ha). European rice is grown under permanently flooded conditions. The irrigation water comes from rivers, such as the Pô in Italy, the Ebro in Spain and the Rhône in France. European rice is partly grown in river flood plains, as in Italy, and partly in river deltas. In river deltas, particularly in areas below sea level, rice soils are prone to high salinity levels. The risk of soil salinization is increasing with observed decreases in river flow due to climate change [2–4] and with the potential development of cropping systems without permanent flooding such as the alternate wetting and drying system aimed at economizing water [5–7]. Although soil salinity can be maintained at manageable levels using water management techniques to leach salt from topsoil [8–9], the use of rice varieties that are tolerant to salinity has been considered as a protective option.

Relative to other cereals, such as barley and wheat, rice is not a crop that is very tolerant to salinity. Maas and Hoffman [10] classified rice as a sensitive species. A reduction in yield is anticipated when the soil electrical conductivity reaches 2–3 dS m^-1^ [11], and a 12% reduction in yield occurs per each dS m^-1^ beyond this threshold [12]. Although there are known tolerant rice accessions, such as Nona Bokra, Pokkali and Hasawi, these accessions are in limited number among *O*. *sativa* rice genetic resources [13–14]. Tolerant accessions are found in all varietal groups but most of these accessions, which often originate from tidal wetlands and mangrove areas, belong to the indica subspecies and, to a significant extent, to the aromatic group [14]. Tolerance to salinity is required at the seedling and reproductive stages, which are the two stages of maximum plant susceptibility [15]. Nona Bokra and Pokkali exhibit tolerance at the seedling stage, while Hasawi is tolerant at the reproductive stage (RK Singh, International Rice Research Institute (IRRI), personal communication). Apparently, the tolerance mechanisms and the genetic control of tolerance are different at the two stages [16].

The mechanisms involved in the response to salinity in rice have been well described [13, 15, 17]. Two stress phases are generally differentiated: an early brief osmotic stress phase, and a subsequent longer-lasting ionic stress phase. In response to exposure to salinity, the Na^+^ sensing and signaling process from roots to shoot rapidly induces a decrease in water uptake, plant growth and transpiration rate. The decrease in water uptake is mediated by abscisic acid (ABA) but whether this decrease is also due to differences in osmotic pressure is unclear [18]. After this first shoot-ion independent response, if the stress persists, Na+ ions migrate to the roots, either passively through an apoplastic pathway in which Na+ ions directly enter the transpiration stream by leakage [19, 20] or actively through a symplastic pathway mediated by cation channels/transporters, such as the high-affinity K^+^ transporter (*HKT*) gene family. The apoplastic pathway is predominant in rice [13, 19]. The influx of salt into the roots activates perception and signaling, which limits further uptake of Na^+^ through the activation of discriminating ion transport systems with high affinities for K^+^ and/or Ca^2+^ but low affinities for Na^+^. The preferential K^+^ and/or Ca^2+^ uptake reduces the transport of Na^+^ from roots to shoots. A low Na^+^/K^+^ ratio is therefore generally considered as a relevant indicator of salinity tolerance, although a recent study questioned its importance [18]. A high Na^+^ concentration in the apoplastic solution activates protection mechanisms that maintain cell turgor under hyperosmotic conditions, particularly by the accumulation of compatible solutes in the cytoplasm, such as proline [13] or trehalose [21] in rice. However, this mitigating response is metabolically costly.

The accumulation of Na^+^ at toxic levels in the leaves induces chlorosis, senescence, necrosis, and ultimately premature death. Potential mitigation mechanisms that enable a reduction of Na^+^ in the shoots while maintaining high cellular K^+^ levels have been described [13]. One of these mechanisms is vigorous growth to dilute salt concentrations in the tissues [22]. This tolerance mechanism is possibly present in Nona Bokra, which develops a huge biomass. Another mechanism to avoid rapid Na^+^ accumulation is sodium exclusion from the transpiration stream, which occurs by sequestrating Na^+^ into the older leaves [23]. Another mitigation mechanism is ion compartmentation into cell vacuoles, which is mediated by Na^+^/H^+^ antiporters, such as those from the vacuolar Na+/H+ antiporter (*OsNHX*) family [24]. A final mechanism is scavenging of phytotoxic reactive oxygen species (ROS) produced under stress by the activation of antioxidant enzymes, such as ascorbate peroxidase or peroxide dismutase, which limits membrane injuries and protects chloroplast function [13]. Although some authors have indicated that none of these mechanisms are preferentially used in rice [18], other authors have suggested that tissue tolerance mechanisms play secondary roles [14].

When the traits that may be affected by the salinity stress are assessed irrespective of the involved mechanisms (i.e., in the whole-plant perspective), vegetative growth is reduced. Shoots are more affected than roots [13], which in turn increases the root-to-shoot ratio. Cell expansion in young leaves is affected, decreasing the specific leaf area [25]. The thickening of leaf cell walls is sometimes considered adaptive because this process provides a greater volume in which salt can be sequestered [26]. Specific leaf area linearly increases with increasing relative growth rate [25]. In addition, at a later stage, salt stress induces delayed panicle initiation and flowering. Yield components, notably spikelet fertility, are affected by Na^+^ accumulation in flag leaves and developing panicles [26, 27].

The genetic control of salt tolerance is only partially understood. Because of the diversity of the involved mechanisms, the genetic architecture of salt tolerance is complex. Numerous quantitative trait loci (QTLs) have been identified using bi-parental mapping populations (reviewed by Negrao et al. [13] for studies conducted prior to 2011, [28–29]), and, more recently, association mapping panels [12, 30–33]. Lists of genes involved in salinity tolerance based on the study of mutants or transgenic plants have been established [13, 34]. The gene *OsHKT1;5*, also called *SKC1*, which encodes a Na^+^-selective transporter, has been implicated in the tolerance of Nona Bokra [35]. A major QTL from Pokkali, named Saltol, which is involved in Na/K homeostasis, has been fine mapped in the same area of chromosome 1, but genes from this segment other than *OsHKT1;5* may be implicated as well [36–37]. Pokkali carries the same haplotype as Nona Bokra at *HKT1;5* [38]. The tolerance allele of *HKT1;5* may originate from the aromatic groups [14]. Pokkali and Nona Bokra have been largely used as donors in breeding programs for the marker-assisted selection of salinity tolerance and varieties as tolerant as the donor parent, such as BRRI Dhan 47, have been released [39]. Although excellent results were obtained by introgressing the Saltol QTL into mega-varieties [36, 39–41], this QTL alone is not sufficient to obtain a high degree of salt tolerance under all conditions and stages. A pyramiding approach was proposed several decades ago [22] and Ahmadi et al. [30] showed that even for tolerance at the vegetative stage, the crossing design might involve a large number of accessions that carry different tolerance mechanisms. The question as to which QTLs should be combined must be resolved within each genetic background.

In Europe, because of the risk of low temperatures during the cropping season and the need for accessions to flower during the long days of European summers, almost all rice varieties belong to the temperate japonica group [42]. Thus far, few studies have focused on these accessions, although some studies have evaluated temperate japonicas from other areas [12, 29, 43]. An association study targeted to specific regions of the genome that carry known tolerance genes was performed [30] but no genome-wide association studies (GWASs) have been conducted thus far. The two objectives of the present study are to evaluate the salinity tolerance at the seedling stage of a panel of 240 temperate japonica rice accessions jointly established by breeders from France, Italy and Spain, and to identify potential targets and candidate genes for marker-assisted breeding using genome-wide association mapping.

## Materials and methods

### Plant materials

The panel comprised 240 temperate japonica accessions. The list of all accessions and their countries of origin is provided in S1 Table. The majority of these lines belong to the panel described in Biscarini et al. [44]. The seeds of these accessions were obtained from the Rice Research Unit of the Consiglio per la ricerca in agricoltura e l'analisi dell'economia agraria (Vercelli, Italy). Seeds from additional Spanish and French lines were obtained from the Instituto de Investigación y Tecnología Agroalimentaria (Torre Marimon, Spain) and from the Centre Français du Riz (Arles, France), respectively. Several accessions known for their susceptibility or resistance to salinity were added to the panel. The indica accessions Nona Bokra, Pokkali, BRRI Dhan 47, FL478, IR64-Saltol, and Hasawi were used as tolerant checks. The recombinant inbred lines BRRI Dhan 47 and FL478 derived from the cross IR29 x Pokkali are recognized for their high salinity tolerance [36]. IR64-Saltol is a version of IR64 developed by the International Rice Research Institute (IRRI) in which the Saltol QTL from Pokkali on chromosome 1 was introgressed by marker-aided selection. IR29 (indica), Giano and Aychade (temperate japonica) were used as susceptible checks. All indica lines were obtained from IRRI.

### Genotyping of the panel

The genotyping-by-sequencing (GBS) data used in the present study were derived from pooling the sequences from two batches of accessions (batch 1 and batch 2) obtained from separate libraries. DNA extraction for batch 1 and batch 2 was performed as described in Biscarini et al. [44] and Courtois et al. [45], respectively. Library production and sequencing were conducted in 2013 for batch 1 and in 2016 for batch 2; however, in both cases, the procedures were performed at the Genomic Diversity Facility of Cornell University according to the method of Elshire et al. [46]. The libraries were built using ApeKI for genome digestion. The libraries were sequenced with an Illumina Genome Analyzer II (San Diego, California, USA). The fastq sequences from the two batches were pooled together and aligned to the rice reference genome (Os-Nipponbare-Reference-IRGSP-1.0 [47]). Single nucleotide polymorphism (SNP) calling was performed using the Tassel GBS pipeline v5.2.29 [48]. Five accessions with a rate of missing data above 30% were discarded. Subsequently, markers with a call rate below 80%, a heterozygosity rate above 10% or a minimal number of variant accessions below 5 (corresponding to a minor allele frequency (MAF) < 2.0% in this panel) were discarded. The remaining heterozygotes were converted to missing data. The missing data were imputed using Beagle v4.0 [49]. The final resulting matrix involves 235 individuals and 30 314 markers and can be downloaded in HapMap format from http://tropgenedb.cirad.fr/tropgene/JSP/interface.jsp?module=RICE study “Genotypes”, study type “GreenRice_panel_GBS_data”.

### Phenotyping the panel for salinity tolerance

#### Experimental design

The experimental design of the trial was a split-plot with three replicates staggered over time. The whole plot main factor was salinity treatment (control (CTRL) versus salt (SALT)) while the secondary split-plot factor was genotype. In each replicate, the plants were distributed into 12 tanks (6 control and 6 salted). The tanks were considered sub-plots. Two resistant checks (Nona Bokra and Pokkali) and three susceptible checks (IR29, Aychade and Giano) were replicated in each tank.

#### Plant growth conditions

The experiment was conducted in a growth chamber with 28–24°C day-night temperatures, an 11–13 h day-night photoperiod and a relative humidity of 65%. Tanks of 80 l capacity were used. The tanks were filled with Hoagland and Arnon solution (50 l per tank) [50], pH 5.5, modified as indicated by Courtois et al. [45]. The solution was continuously and gently stirred-aerated using small aquarium pumps (one per tank). The solution was changed every week. The pH was measured every day and adjusted if necessary. Each tank contained a 55.5 cm x 36.6 cm x 3.0 cm foam polystyrene plate with 48 holes drilled at a diameter of 2.5 cm. One hole was left empty and was used for the manipulation of the probe for pH control. Four seeds per accession were sown in the holes of the polystyrene plate. For the first week, a mosquito net with a small mesh was fixed to the bottom of the plate to maintain the seeds. One week after sowing, the polystyrene plates were changed. The most developed seedling of each hole was wrapped in a small 2.5 cm x 2.5 cm x 2.5 cm polyethylene terephthalate fiber cube (Hail mini cubes, Sure to Grow, Ohio, USA) that was cut diagonally through the center. The seed and its attached roots were set below the cube. The wrapped plant was transferred to a new plate without a plastic grid (one plant per hole). Salt treatment was initiated at two weeks after sowing. To limit the osmotic stress, half of the target salt quantity was added to the stressed tanks on day 14. The other half of the target salt quantity was added on day 15. The final concentration was 50 mM NaCl (3 g/l), corresponding to an electrical conductivity of 6.0 dS m^-1^. The salt concentration was selected based on the results of Ahmadi et al. [30] to limit lethality while strongly decreasing the growth of the most susceptible accessions. The plants were grown for two weeks under these conditions. The conductivity was adjusted as needed by adding osmosed water. The mortality percentage was assessed at each change of the solution. Four weeks after sowing, the plants were harvested.

#### Traits evaluated

The effect of the stress on the plant growth rate was measured (Table 1). For each plant, the number of tillers (TIL) was counted. The lengths of the longest leaf (LL) and the longest root (RL) were measured. The blade of the last fully developed leaf was collected, and its length (LGTH) and width (WDTH) were measured. The area of the blade of the last fully developed leaf (LA) was determined as LGTH x WIDTH x 0.75 based on Yoshida's formula [51]. The shoots and roots of each plant were separated and the shoots, roots and last fully developed leaves were oven dried at 72°C for 4 days, after which the dry matter was weighed (SHOOT, ROOT and LEAF). The specific leaf area (SLA) was calculated by dividing LA by LEAF. The root-to-shoot ratio (R/S) was calculated by dividing ROOT by SHOOT.

**Table 1:** Recorded traits with their abbreviations.

Trait name	Unit	Abbreviation
**Number of tillers**		TIL
**Maximum leaf length**	cm	LL
**Maximum root length**	cm	RL
**Root dry weight**	g	ROOT
**Shoot dry weight**	g	SHOOT
**Last fully developed leaf dry weight**	g	LEAF
**Root-to-shoot ratio**		R/S
**Leaf area**	cm^2^	LA
**Specific leaf area**	cm^2^g^-1^	SLA
**Sodium mass fraction**	%	Na
**Potassium mass fraction**	%	K
**Sodium-to-potassium ratio**		Na/K

The whole shoot dry mass of the stressed set of accessions was cut into small pieces and subsequently ground to a powder using a mechanical grinder (one 5 mm-diameter bead per tube; 2 times for 1 min at 1400 rpm). Then, the Na and K mass fractions (Na and K), expressed as a percent of dry matter, were measured for each sample by atomic emission spectroscopy (ICP-AES). The analyses were conducted at the UR59 laboratory in Cirad Montpellier, France (ISO 9001). The Na/K ratio was calculated by division. In addition, several check plants from the unsalted treatment (3 plants of Nona Bokra and 3 plants of IR29 for each replicate) were treated in the same manner. The data are available in S1 Table.

### Statistical analyses

Analysis of variance was conducted on the growth parameters using a mixed model with treatment and genotype as fixed effects, and replicate and tank as random effects. The significance of genotype, treatment and genotype x treatment interaction effects were tested. The least square (LS) mean values were calculated and adjusted from the tank effect. For each measured growth trait, considering intrinsic differences in growth rate between accessions, indices of response to stress were calculated based on the LS mean values as iTRAIT = (SALT—CTRL) x 100 / CTRL. Such indices can be strongly affected by the poor growth of some control plants. A Grubbs’ test (P < 0.05) was therefore conducted to remove the outliers using XLStat [52]. For the ions measured only under stressed conditions, the analysis of variance was conducted without treatment effects. Broad-sense heritabilities at the genotypic mean level were calculated for each condition and trait by running a separate analysis of variance without treatment effects with h^2^ = σ^2^G/(σ^2^G+σ^2^e/n), where σ^2^G and σ^2^e are the estimates of genetic and residual variances, respectively, derived from the expected mean squares of the analysis of variance and n is the number of replicates. The heritabilities of the indices were obtained in the same manner after computing the indices for each replicate. Since some of the measured traits were correlated, a principal component analysis (PCA) was conducted on all traits using XLStat.

### Genome-wide association study

A GWAS was conducted on the 231 japonica accessions in the panel having both phenotypic and genotypic data using two different methods, termed the “classical method” and the “interaction method”. For the classical method, the GWAS analyses were conducted using a mixed linear model (MLM) with control of panel sub-structure (fixed effect) and kinship among accessions (random effect) using Tassel v5 [53]. The coordinates of the accessions in the two first axes of a PCA conducted on the genotyping data were used to control the population structure. The number of axes was set to two because previous results from an analysis of population structure showed that the main differentiation among European accessions was observed between temperate and tropical-derived accessions [42]. The kinship matrix was calculated using the centered identity by state method, which provides a matrix equivalent to the VanRaden matrix. As described by Courtois et al. [45], a comparison was first conducted between models with kinship alone and kinship and structure using the Akaike information criterion (AIC). The AIC was calculated as -2 ln(L) + 2k, where ln is the natural logarithm, L is the maximized value of the likelihood function for the estimate model and k is the number of estimated parameters [54]. This comparison showed that the second model best fit the data (S2 Table and S3 Fig) and this model was adopted for further analyses. To determine significance and ensure the robustness of the detected associations, a sub-sampling procedure was conducted, according to Tian et al. [55] (maize), and Lafarge et al. [56] and Bettembourg et al [57] (rice). A set of 200 accessions (approximately 80% of the panel size) was selected at random without replacement and a GWAS was conducted on this set. The random sub-sampling and GWAS analysis were repeated 100 times. The number of times an association was detected at P < 0.001 in the 100 sub-samples was counted to obtain a sub-sampling posterior probability for each marker. Based on the distribution of the results, a threshold that had a 95% chance not to be overtaken was selected separately for each trait. The significances reported in the result table for a given marker are those from the GWAS analysis conducted on the entire panel. Markers that were significant within an interval of less than 100 kb were considered to belong to the same QTL. This window size was selected because it was intermediate between the average marker distance (approximately 12 kb in the GBS dataset) and the distance at which the linkage disequilibrium (LD) decayed to half its initial level (estimated at 400 kb in the panel on average across all chromosomes but much more variable in short-range segments). Quantile-quantile (QQ) plots and Manhattan plots were obtained from Tassel or drawn using the qqman R package.

For traits measured both under control and salt conditions, the interaction method, described in detail in Al-Tamimi et al. [33], was also used. The interaction MLM model includes the main effects of the marker and treatment (control and salt, respectively) and marker x treatment interaction, using the same PCA coordinates and kinship matrix obtained from Tassel. This approach enables the identification of SNPs significant for the marker x treatment term and therefore, the identification of loci that respond to salinity treatment. The scripts from Al-Tamini et al. [33] required ASReml-R [58], a proprietary package for the R environment [59]. In this second case, sub-sampling was not attempted because of the time necessary for analyses with ASReml. A false discovery rate (FDR) of 0.05 was used for determining the significance for all traits [60].

In both cases, once a first list of significant markers was established, an LD matrix among these markers was calculated using Tassel, and pairs of close markers with an r^2^ value above 0.8 and similar MAFs were pooled in the same interval.

### Candidate genes

A list of genes shown to play a role in salt tolerance in rice, referred to as salt tolerance genes, with their identifiers in the two rice gene nomenclatures, physical, position, annotation, function relative to salt stress (when known) and digital object identifier (DOI) of the reference paper(s) was established based on an update of previous lists established by Negrao et al. [13], Kumar et al. [34] and the OGRO database (http://qtaro.abr.affrc.go.jp/ogro). The gene positions and annotations were obtained from Michigan State University (http://rice.plantbiology.msu.edu/), release 7. This list of approximately 300 genes (S2 Table) was used to assess whether the association positions were close to known genes. In addition, all genes with an annotated function in a window of +/-100 kb around the significant markers, or in the interval between two positions when its size was above 200 kb, were extracted using OrygenesDB tools (http://orygenesdb.cirad.fr/), and their annotations were also explored using key words to assess whether these genes had a plausible relationship with salinity tolerance. The gene *HKT1;5* (*Os01g20160*), located in the center of the Saltol QTL, has a recognized importance in salinity tolerance in rice [14]. To assess the diversity of this gene among temperate accessions, a haplotype analysis of the gene was conducted using the sequences of *HKT1;5* from accessions of the 3,000 rice genomes project [61]. The analyzed set comprised the 57 European accessions common to both our panel and the 3,000 genomes project (18 classified as "japx", 30 as "temp" and 9 as "trop" in the IRIC database (http://oryzasnp.org/iric-portal/), with the term "GERVEX" included in their IRGC-ID; listed in S1 Table), 536 random accessions from the 3,000 genomes project, and seven accessions mentioned by Platten et al. [14] as references for the various haplotypes (IR64, Pusa basmati, Pokkali, Nona Bokra, FL478, Nipponbare and Azucena) with known salinity tolerance levels. For these 600 accessions, the sequences involving 76 polymorphic SNPs were extracted from the IRIC database. An unweighted neighbor-joining tree was built using a simple matching index.

## Results

### Response to salinity

After 28 days of growth with two weeks of salinity stress at a conductivity of 6.0 dS.m-1, the hydroponic experiment was harvested. Lethality affected 4.8% of the control plants and 8.0% of the stressed plants with similar proportions in all replicates, indicating a slight increase in mortality due to salt treatment. However, approximately two-thirds of the plants that died were recorded as having poor germination or were scored as being weak prior to treatment (63.4% for the control plants and 64.7% for the stressed plants).

**Fig 1:**
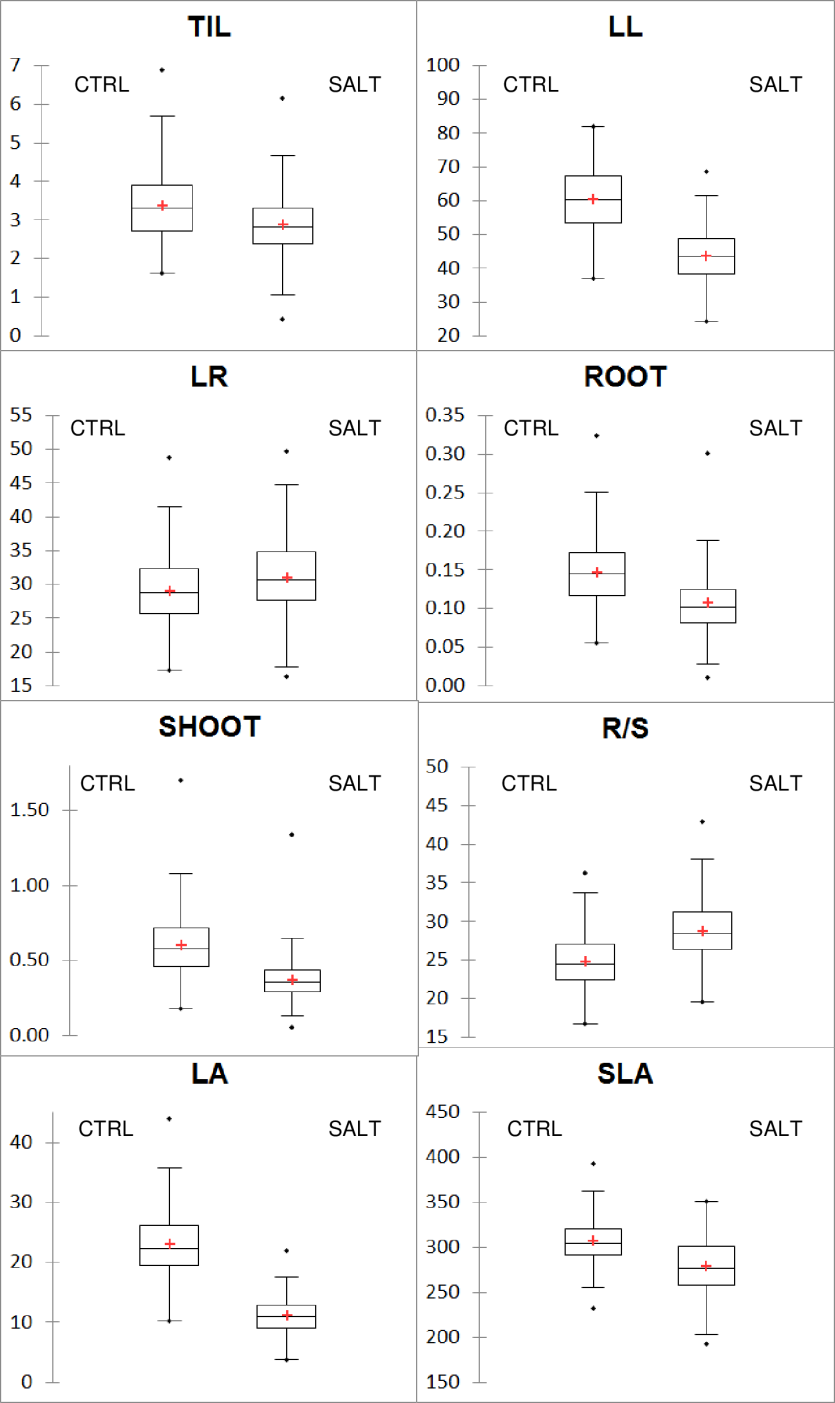
Box-plots of the two treatments for the nine growth traits. CTRL: control treatment; SALT: salt treatment; TIL: number of tillers; LL: maximum leaf length; RL: maximum root length, ROOT: root biomass; SHOOT: shoot biomass; R/S: root-to-shoot ratio; LA: leaf area; and SLA: specific leaf area.

**Fig 2:**
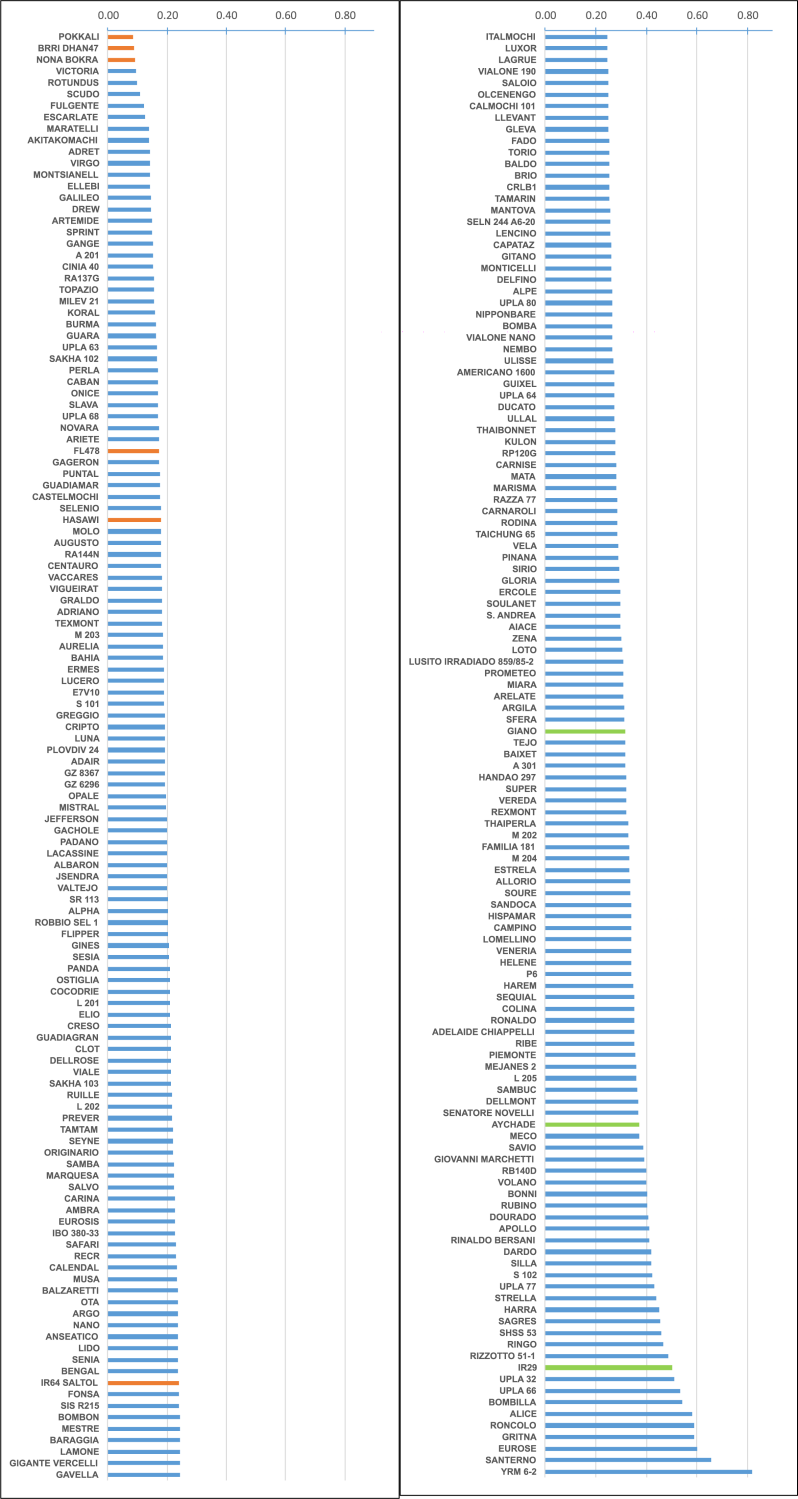
Distribution of the Na/K ratio among the tested accessions. In orange, tolerant checks; in green, susceptible checks; and in blue, temperate japonica accessions.

The ANOVA results showed highly significant treatment and genotype effects and highly significant genotype x treatment interactions for all growth traits except TIL (Table 2). The significant interactions indicated that the accessions responded differently to the salinity treatment and that variability in tolerance levels existed among the tested accessions. On average, the salinity treatment negatively affected all growth traits, except RL and R/S, which were positively affected (Table 3; Fig 1). LA (-51.2%) and SHOOT (-36.5%) were the traits most impacted by salinity stress, followed by LL (-27.0%) and ROOT (-25.7%). Significant differences between accessions were also observed for Na^+^ and K^+^ leaf mass fractions and the Na/K ratio (Table 2). Based on the results of the two extreme checks (Nona Bokra and IR29) in which the ion mass fractions were measured under both control and stressed conditions, the mean increase in Na^+^ leaf content varied from 400% (Nona Bokra) to 2000% (IR29), while the mean decrease in K^+^ leaf content varied only from 35% (Nona Bokra) to 56% (IR29). As shown in Fig 2, which is based on the Na/K ratio, the temperate accessions were regularly distributed over an increasing axis from Nona Bokra, Pokkali and BRRI Dhan 47, the most resistant checks with the lowest Na/K ratio (in orange in Fig 2), to Aychade, Giano and IR29, the most susceptible checks with the highest Na/K ratio (in green in Fig 2). No accession was more tolerant than Nona Bokra under the selected stress conditions, but certain accessions, such as Victoria or Rotundus, were close. A few accessions, such as Santerno or YRM6-2, were more susceptible than IR29. When the checks were excluded, the range of variation in the panel was from 0.240% to 1.387% (6-fold difference) for Na, 1.461% to 3.111% for K (2-fold difference) and 0.095 to 0.819 (9-fold difference) for Na/K.

**Table 2:** Probabilities of the effects tested by ANOVA for the different traits.

Trait	TT	Genotype	TT x Genotype
**TIL**	0.0002	< 0.0001	0.1195
**LL**	< 0.0001	< 0.0001	< 0.0001
**RL**	< 0.0001	< 0.0001	< 0.0001
**ROOT**	< 0.0001	< 0.0001	0.0010
**SHOOT**	< 0.0001	< 0.0001	< 0.0001
**R/S**	< 0.0001	< 0.0001	< 0.0001
**LA**	< 0.0001	< 0.0001	< 0.0001
**SLA**	< 0.0001	< 0.0001	0.0043
**Na**		< 0.0001	
**K**		< 0.0001	
**Na/K**		< 0.0001	

TT: treatment

**Table 3:** Statistical parameters for all traits in the control (CTRL) and salinity (SALT) treatments and for the indices of stress response.

Trait	CTRL	CTRL	CTRL	CTRL	SALT	SALT	SALT	SALT	iTRAIT	iTRAIT	iTRAIT	iTRAIT
	Min	Max	Mean	Stdev	Min	Max	Mean	Stdev	Min	Max	Mean	Stdev
**TIL**	1.61	6.89	3.38	0.91	0.43	6.17	2.90	0.82	-59.8	45.3	-12.2	21.5
**LL (cm)**	36.9	82.0	60.4	8.6	24.3	68.8	43.8	6.9	-45.6	-8.5	-27.0	6.7
**RL (cm)**	17.3	48.8	29.05	5.22	16.39	46.67	31.14	5.65	-34.2	48.8	7.3	15.2
**ROOT (g)**	0.0552	0.3242	0.1471	0.0467	0.0109	0.3008	0.1075	0.0395	-74.3	29.4	-25.7	20.0
**SHOOT (g)**	0.1785	1.7043	0.6043	0.2191	0.0553	1.3406	0.3753	0.1439	-77.8	8.6	-36.5	17.5
**R/S**	16.77	36.23	24.88	3.47	19.6	46.49	28.88	4.07	-12.1	50.8	17.1	13.0
**LA (cm**^**2**^**)**	10.20	43.86	23.04	5.55	3.89	21.95	11.16	2.95	-76.4	-27.1	-51.2	10.2
**SLA (cm**^**2**^ **g**^**-1**^**)**	232	393	307	24	193	351	279	30	-33.1	14.4	-8.8	9.6
**Na (%)**					0.240	1.387	0.577	0.168				
**K (%)**					1.461	3.131	2.306	0.306				
**Na/K**					0.084	0.819	0.266	0.105				

Min: minimum; Max: maximum; Stdev: standard deviation; iTRAIT = (SALT-CTRL) x 100 / CTRL

The heritabilities of the different traits were moderate to high (0.49 to 0.92) when calculated for each treatment, and for a given trait, the heritabilities were of the same magnitude for both treatments (Table 4). However, the response indices registered much lower heritabilities (0.12 to 0.55). This characteristic translated to the lowest reliability of indices due to propagation of uncertainty in functions involving several variables, and this effect occurred even after removing potential outliers with abnormal growth patterns using a Grubbs’ test.

**Table 4:** Broad-sense heritabilities for the different traits in the different treatments.

Trait	CTRL	SALT	Index
**TIL**	0.73	0.75	0.12
**LL**	0.89	0.92	0.55
**RL**	0.79	0.81	0.37
**ROOT**	0.79	0.87	0.38
**SHOOT**	0.83	0.91	0.36
**R/S**	0.82	0.85	0.52
**LA**	0.79	0.81	0.38
**SLA**	0.50	0.49	0.26
**Na**		0.53	
**K**		0.72	
**Na/K**		0.61	

The correlations among indices of growth response to stress were generally high and positive (0.24 to 0.91), except for correlations with iSLA (all negative) and iR/S. The correlation between Na and K was intermediate and negative (-0.39). The correlations between indices of growth response and the Na/K ratio were low and negative; these correlations were significant (-0.23< r <-0.14) for iLL, iROOT, iSHOOT, and iLA, and not significant for iTIL, iR/S and iSLA (S1 Fig), indicating that the phenomena involved in growth response and ion accumulation may be somewhat different. To assess whether the dilution factor played a role in the salinity tolerance of the panel, the correlations between ion-related traits and shoot biomass of the control treatment were calculated; however, the correlations were close to 0 and were not significant. These trends were confirmed by the results of a PCA conducted on the 11 variables. The first two axes explained 57.6% of the variation. The indices of stress response were the main contributors to axis 1, while the ion mass fractions were the main contributors to axis 2 (S2 Fig).

### Genome-wide associations

The indica checks were excluded from further analyses, and GWAS analyses were conducted on the 231 accessions that had genotypic data. The eight stress response indices and the three ion parameters were analyzed. The results of the analyses are presented in Table 5 for the classical method and Table 6 for the interaction method. In this narrow genetic base-panel, LD slowly decays. To assess whether the markers, consecutive or not, corresponded to different QTLs, the LD between significant markers was calculated for both methods (S3 Table). No long range or cross-chromosome correlations between significant markers were observed. However, in a few cases, close markers with similar MAFs were found in strong LD (r^2^ above 0.8) and grouped in the same interval. The limits of these intervals, which exceptionally exceeded 500 kb, are indicated in Tables 5 and 6.

**Table 5:** Significant markers in the GWAS using the classical method.

QTL	Chr	Pos1	Pos2	Size	MA	iTIL	iLL	iRL	iROOT	iSHOOT	iLA	iSLA	iR/S	K	Na	Na_K
**q01_01**	1	717 021	879 846	163	35					2.49E-04						
**q02_01**	2	9 619			77		1.43E-04	2.55E-04							2.16E-04	4.76E-05
**q02_02**	2	10 143 911	14 415 077	4 271	9				5.76E-05							
**q02_03**	2	35 313 501			36									2.95E-04		
**q03_01**	3	13 477 700			6								4.84E-04			
**q03_02**	3	15 563 875	15 685 034	121	112						3.33E-04					
**q03_03**	3	15 697 407	16 253 524	556	82							3.77E-05				
**q03_04**	3	20 480 219			109					2.26E-04						
**q04_01**	4	2 476 275	2 480 523	4	14									2.71E-04		
**q04_02**	4	19 300 224			42		1.36E-04									
**q06_01**	6	895 128			8								3.28E-04			
**q06_02**	6	4 405 810	6 063 085	1 657	39						5.31E-05					
**q06_03**	6	21 981 979	21 982 001	1	106	5.47E-05										
**q06_04**	6	26 302 723			56		1.20E-04									
**q07_01**	7	7 046 395			9											3.47E-04
**q07_02**	7	22 274 975			83		9.13E-05									
**q08_01**	8	3 822 046	3 822 071	1	9											4.37E-04
**q08_02**	8	7 160 262			9										3.42E-04	3.43E-06
**q08_03**	8	27 352 942	27 354 916	2	8									3.96E-04		6.93E-06
**q09_01**	9	3 783 034	5 176 111	1 393	100					1.58E-04						
**q09_02**	9	6 580 479			91								1.15E-04			
**q09_03**	9	10 323 934			98					1.43E-04						
**q09_04**	9	12 067 716			95			4.22E-04								
**q10_01**	10	21 117 030	21 174 348	57	9											4.89E-04
**q11_01**	11	20 874 275			9								4.95E-04			
**q11_02**	11	21 246 644	21 246 751	1	22									4.69E-04		
**q11_03**	11	27 799 249			34	2.24E-04										
**Nb of QTLs**		** **	** **	** **	** **	**2**	**4**	**2**	**1**	**4**	**2**	**1**	**4**	**4**	**2**	**6**

Chr: chromosome; Pos1-Pos2: limits (bp) of the interval with a LD>0.8; Size: size of the interval in kb; MA: number of accessions carrying the minor allele; iTIL: relative number of tillers; iLL: relative maximum leaf length; iRL: relative maximum root length, iROOT: relative root biomass; iSHOOT: relative shoot biomass; iLA: relative leaf area; iSLA: relative specific leaf area; and iR/S: relative root-to-shoot ratio.

**Table 6:** Significant markers in the GWAS using the interaction method.

QTL	Chr	Pos1	Pos2	Size	MA	TIL	RL	ROOT	SHOOT	LA	SLA
**q01_01**	1	717 021			37			3.97E-05			
**q01_02**	1	2 228 168			97				3.97E-05		
**q01_03**	1	7 015 229			45			2.64E-05	1.35E-05		
**q01_04**	1	12 176 520			71					2.25E-07	
**q01_05**	1	24 026 841			5	7.95E-06					
**q01_06**	1	24 984 212	24 984 220	1	83						2.55E-06
**q01_07**	1	26 424 572	26 588 934	164	62						8.94E-07
**q01_08**	1	29 154 215	29 203 007	49	46						7.73E-07
**q01_09**	1	42 986 168			57					1.45E-07	
**q02_04**	2	10 595 315			95			2.69E-05			
**q02_05**	2	21 847 001	21 858 276	11	105		1.52E-07				
**q02_06**	2	22 114 658	23 678 820	1 564	31					1.30E-07	
**q02_07**	2	24 553 256			110		3.85E-06				
**q02_08**	2	25 445 687	25 445 690	1	59			2.55E-05			
**q02_09**	2	25 885 060	25 990 477	105	99		8.57E-07				
**q02_10**	2	28 113 111			39					2.00E-07	
**q02_11**	2	28 486 697	29 118 703	632	28			8.64E-06	1.75E-05		
**q03_05**	3	270 110			64						2.88E-07
**q03_06**	3	28 101 825	28 141 030	39	26					6.21E-07	
**q04_03**	4	20 318 757			7	2.17E-06		5.16E-06	1.44E-05		
**q04_04**	4	21 610 862			115		4.89E-06				
**q04_05**	4	29 922 354	29 922 382		69					8.58E-07	
**q04_06**	4	30 482 527	30 482 534	1	43				4.82E-05		
**q04_07**	4	31 707 318			30				6.30E-06		
**q04_08**	4	32 277 704			10				6.24E-06		
**q05_01**	5	3 615 491	3 615 493	1	11			1.50E-05	1.73E-05	8.51E-07	
**q05_02**	5	4 771 178			9					3.54E-08	
**q05_03**	5	5 025 167	5 025 170	1	7			1.92E-05	9.03E-06	1.11E-07	
**q05_04**	5	21 791 187			13			6.87E-06			
**q05_05**	5	23 572 890	23 593 730	21	9			6.38E-06	8.57E-06	3.10E-07	
**q06_02**	6	4 405 810	6 063 085	1 657	40					9.64E-08	
**q06_05**	6	18 153 220	18 191 644	38	93					3.19E-08	
**q06_06**	6	21 539 402	21 552 746	13	111						2.01E-06
**q06_07**	6	21 802 147	22 225 475	423	53		2.32E-06		1.46E-05	2.71E-09	
**q06_03**	6	21 981 979	21 982 001	1	107	4.38E-07					
**q07_03**	7	17 817 832	19 631 592	1 814	6			2.71E-06	2.05E-06		
**q07_04**	7	22 931 083	23 068 438	137	13			2.43E-05			
**q08_04**	8	750 753			33	4.54E-05					
**q08_05**	8	21 353 911	21 466 673	113	110						5.62E-08
**q08_06**	8	21 851 814			69						1.49E-08
**q09_05**	9	18 387 915			33					8.66E-07	
**q09_06**	9	21 329 392			22				3.87E-05		
**q10_02**	10	15 211 421			15			3.34E-05	1.71E-06		
**q10_03**	10	20 065 884	20 144 369	78	51						3.49E-06
**q10_04**	10	21 618 949	21 893 599	275	13		3.75E-05				
**q11_04**	11	4 376 870			67						2.18E-06
**q11_05**	11	22 302 306			22					7.42E-07	
**q12_01**	12	17 940 089			106	2.88E-05					
**q12_02**	12	20 014 218	20 789 916	776	104		3.10E-07				
**q12_03**	12	21 834 586			34		3.11E-07				
**Nb of QTLs**						5	8	13	14	15	9

Chr: chromosome; Pos1-Pos2: limits (bp) of the interval with LD > 0.8; Size: size of the interval in kb; MA: number of accessions carrying the minor allele; TIL: number of tillers; LL: maximum leaf length; RL: maximum root length, ROOT: root biomass; SHOOT: shoot biomass; R/S: root-to-shoot ratio; LA: leaf area; and SLA: specific leaf area.

**Fig 3:**
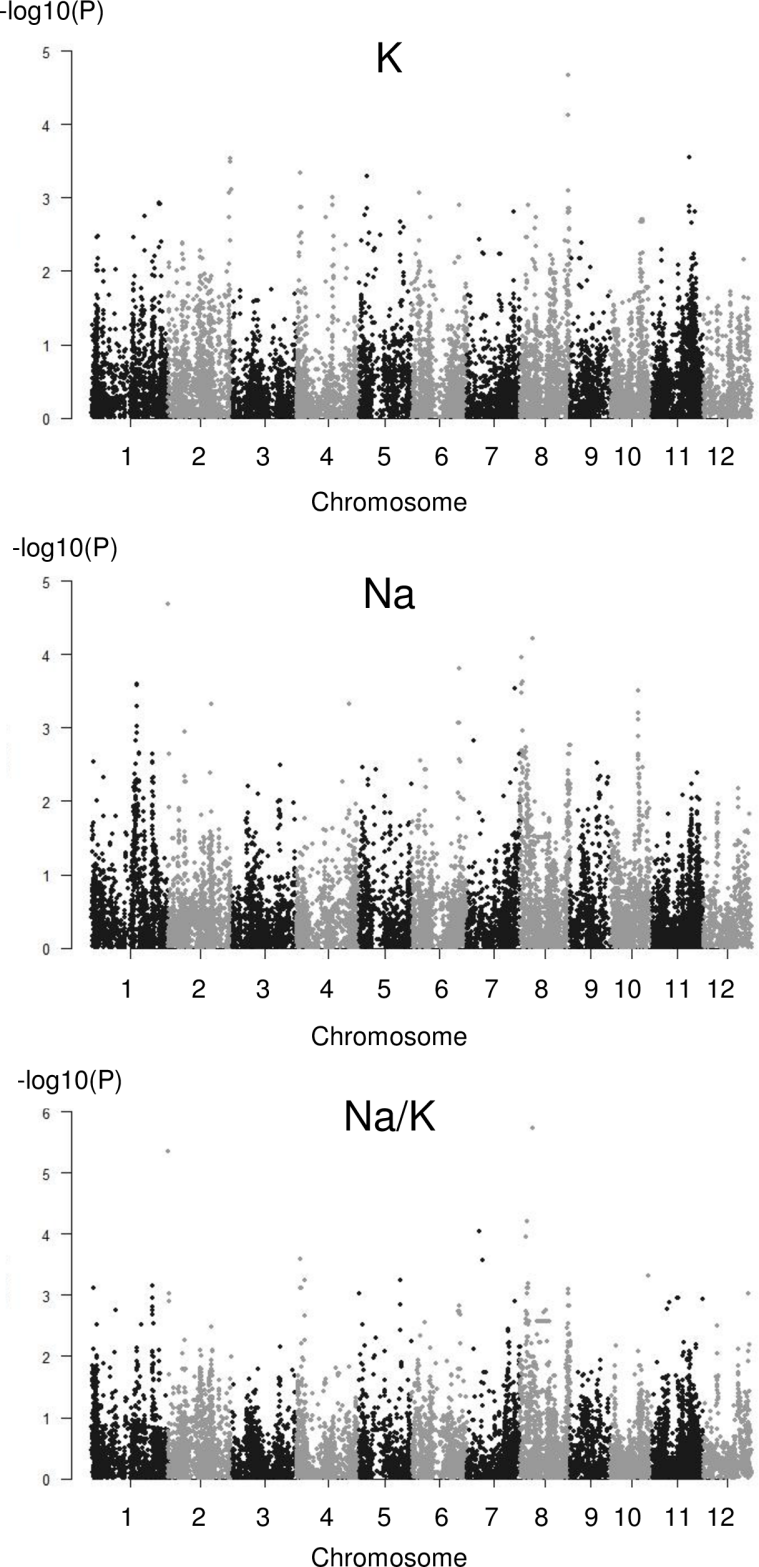
Manhattan plots for Na, K and Na/K using the classical GWAS method.

For the classical method, a total of 27 independent significant associations validated by sub-sampling were identified, with the number per trait varying from 1 to 6. Fewer associations were generally detected for the indices of stress response than for the ion concentrations, which is likely due to the lower heritability of the indices than that of the directly measured parameters. The significance was generally moderate (P > 1.0E-06). One association was common between four traits (iLL, iRL, Na and Na/K), and two others associations were common between two traits (Na and Na/K and K and Na/K). The Manhattan plots corresponding to the ions traits are presented in Fig 3 and those for the indices of response are presented in S4 Fig.

**Fig 4:**
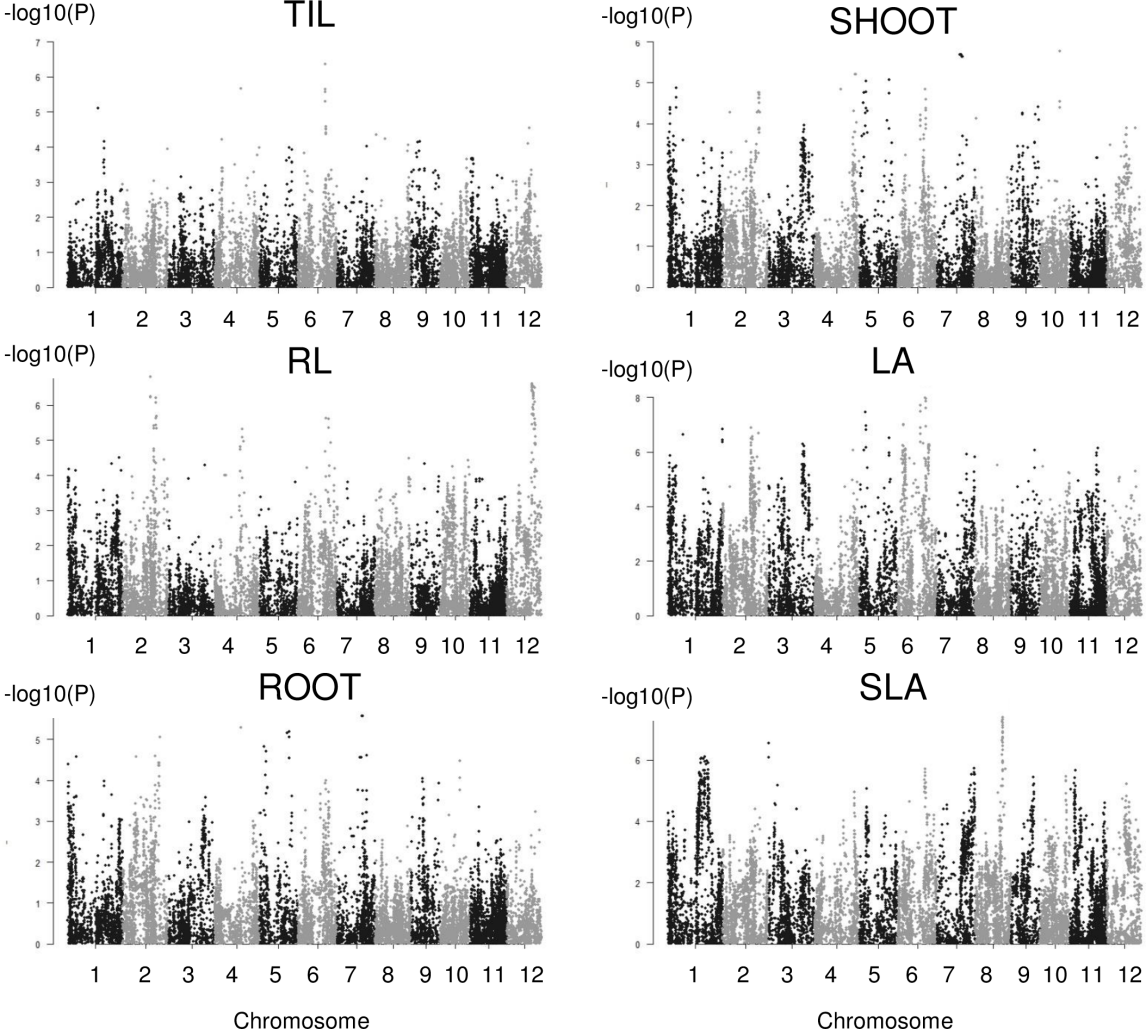
Manhattan plots for the six traits analyzed with the interaction method for which significant associations were detected.

For the interaction method (Fig 4), a total of 50 significant associations corresponding to qvalues below 0.05 were identified with the number per trait varying from 0 (no QTL detected for LL and R/S) to 15; this range is larger than the range described for the classical method. The significance was also much higher (up to P = 1E-09). More associations were common between traits (four between two traits and five between three traits). These common associations were primarily found between highly correlated traits. Three associations (q01_01, q06_02, and q06_03,) were detected using both methods, among which two associations were detected for the same traits in both methods (q06_02 and q06_03).

**Fig 5:**
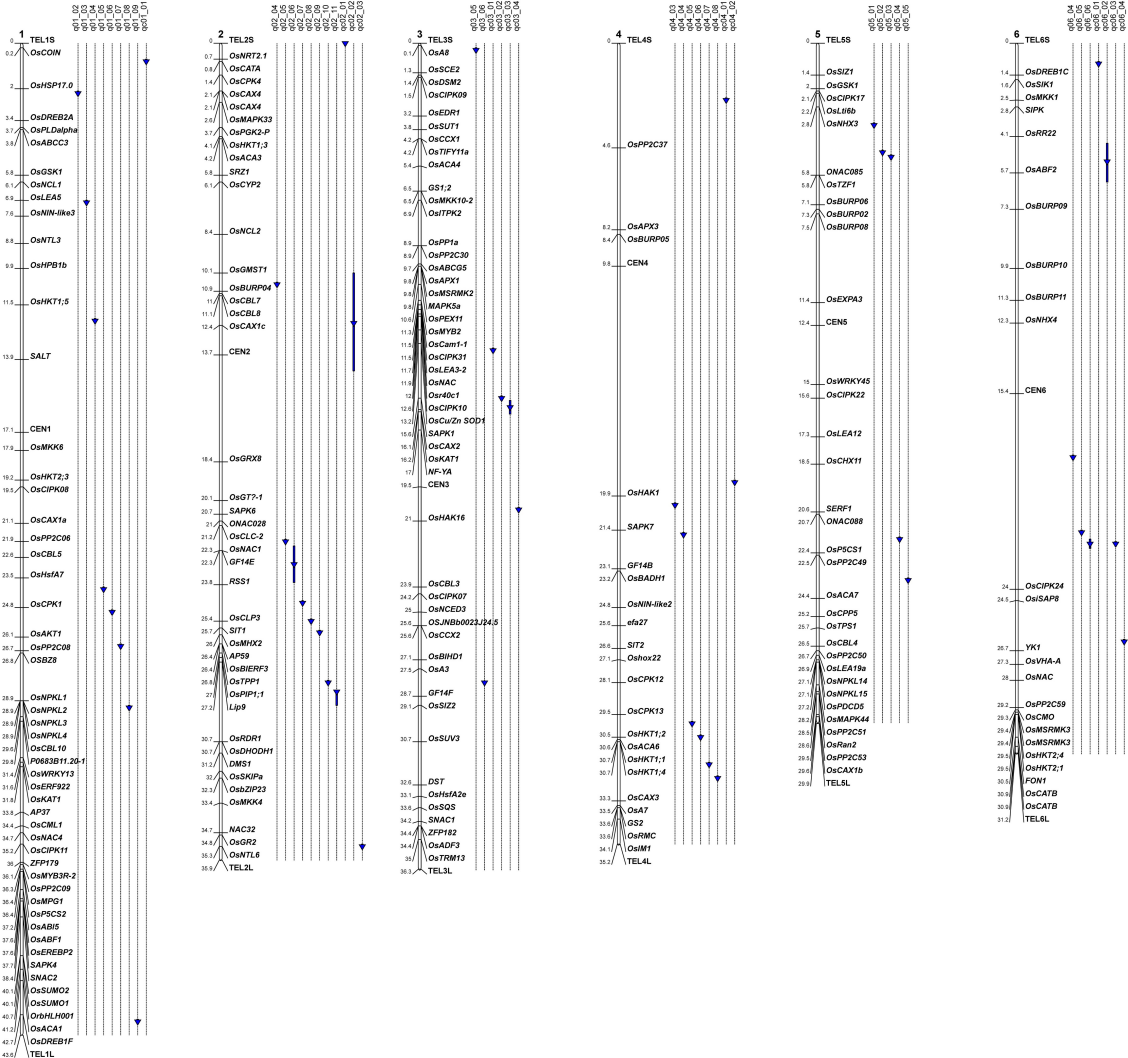
Map of the detected associations compared to the positions of genes involved in salinity tolerance in rice. Chromosomes 1 to 6.

**Fig 6:**
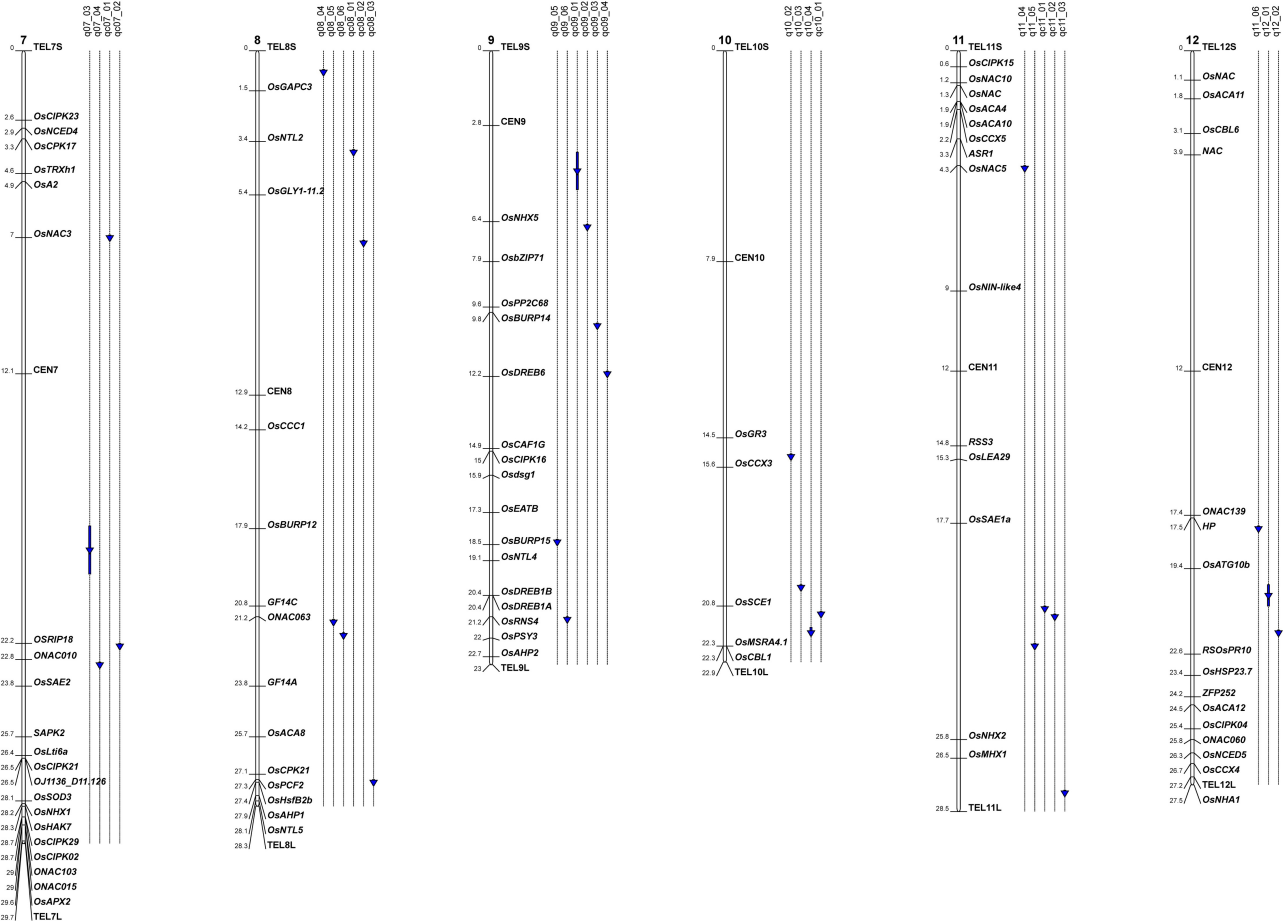
Map of the detected associations compared to the positions of genes involved in salinity tolerance in rice. Chromosomes 7 to 12.

The positions of all significant markers were compared to those of the salt tolerance genes listed in S2 Table (Fig 5 for chromosomes 1 to 6 and Fig 6 for chromosomes 7 to 12). A salt tolerance gene was found in the vicinity (< 100 kb) of 23 of the significant markers (13 markers for the classical method and 10 markers for the interaction method). The list includes several ion transporters, notably a sodium transporter (*OsHKT1;2*), and several genes involved in Ca^2+^ signaling or metabolism, including several protein kinases (Table 7). Based on a key word search, the exploration of 1676 genes with tentative functions found in the same intervals (S4 Table) enabled the identification of 84 additional candidates (highlighted in blue in S3 Table). These candidates included 14 antioxidants, 31 transporters, 15 phosphatases, 5 calcium/calmodulin binding proteins, 3 LEA and 3 aquaporins. The remaining candidates were the sole representatives in their broad functional classes.

**Table 7:** List of salt tolerance genes located less than 100 kb from the significant markers.

QTL	Trait	Chr.	Start	End	Gene	Symbol	Distance	Gene function
**qi01_03**	ROOT	1	6.922	6.923	Os01g12580	OsLEA5	92 kb	Late embryogenesis abundant protein.
**qi01_06**	SLA	1	24.839	24.844		OsCPK1	95 kb	Serine/threonine protein kinase with calmodulin-like domain. Calcium sensor.
**qc02_02**	iROOT	2	10.897	10.902	Os02g18690	OsBURP04	In	Unknown function.
**qc02_02**	iROOT	2	11.018	11.016	Os02g18880	OsCBL7	In	Calcium sensor interacting with CIPK serine-threonine protein kinases.
**qc02_02**	iROOT	2	11.060	11.058	Os02g18930	OsCBL8	In	Calcium sensor interacting with CIPK serine-threonine protein kinases.
**qc02_02**	iROOT	2	12.432	12.449	Os02g21009	OsCAX1c	In	Sodium/calcium exchanger protein playing a role in intracellular Ca^2+^ ion homeostasis.
**qi02_06**	LA	2	22.259	22.261	Os02g36880	OsNAC1	In	NAC-type DNA-binding protein.
**qi02_06**	LA	2	22.333	22.338	Os02g36974	GF14E	In	Regulator of cellular signal transduction.
**qi02_08**	ROOT	2	25.431	25.433	Os02g42290	OsCLP3	12 kb	ATP-dependent clp protease with established stress protective role.
**qi02_09**	RL	2	25.959	25.957	Os02g43110	OsMHX2	8 kb	Role in intracellular Ca^2+^ ion homeostasis.
**qc02_03**	K	2	35.303	35.307	Os02g57650	OsNTL6	10 kb	Membrane bound NAC-type TF. Role in adaptation to osmotic stress.
**qc03_02**	iLA	3	15.632	15.628	Os03g27280	SAPK1	In	Response to hyperosmotic stress.
**qc03_03**	iSLA	3	16.061	16.065	Os03g27960	OsCAX2	In	Role in intracellular Ca^2+^ ion homeostasis.
**qc03_03**	iSLA	3	16.167	16.164	Os03g28120	OsKAT1	In	Potassium channel protein. Maintenance of cytosolic cation homeostasis.
**qi04_06**	SHOOT	4	30.548	30.546	Pseudogene	OsHKT1;2	63 kb	Na^+^ transporter. Pseudogene in Nipponbare.
**qi04_06**	SHOOT	4	30.584	30.572	Os04g51610	OsACA6	90 kb	Calcium-transporting ATPase, plasma membrane-type. Antioxidant.
**qc06_02**	iLA	6	5.677	5.682	Os06g10880	OsABF2	In	Transcriptional regulator modulating expression of salt stress-responsive genes through an ABA-dependent pathway
**qc07_01**	Na//K	7	6.968	6.969	Os07g12340	OsNAC3	78 kb	NAC-type TF.
**qc07_02**	iLL	7	22.223	22.222	Os07g37090	OSRIP18	51 kb	Potential superoxide dismutase activity.
**qc08_03**	K, Na/K	8	27.298	27.296	Os08g43160	OsPCF2	55 kb	TF regulating OsNHX1 expression.
**qc08_03**	K, Na/K	8	27.390	27.381	Os08g43334	OsHsfB2b	28 kb	Heat shock factor.
**qi09_05**	LA	9	18.457	18.459	Os09g30320	OsBURP15	69 kb	Unknown function.
**qi11_04**	SLA	11	4.302	4.299	Os11g08210	OsNAC5	75 kb	NAC-type DNA-binding protein.

Chr.: chromosome; Start-End: Position of the gene in Mb; Distance: distance from the nearest significant marker. Gene symbols and functions refer to the gene list and functions shown in S2 Table.

No associations near the Saltol QTL, located between 10.8 and 11.8 Mb on chromosome 1 and centered on the *HKT1*;5 gene [36], were significant. The analysis of *HKT1;5* polymorphisms of the 57 European temperate accessions in the present panel that were also included in the 3,000 genomes project database showed that the temperate accessions carried haplotypes belonging to closely related branches of the tree. All haplotypes belonged to clusters in which the reference accession did not carry tolerance alleles for this gene (S5 Fig). It is therefore likely that there is no significant association in this zone because no temperate accession carries *HKT1;5* tolerant haplotypes.

## Discussion

We conducted hydroponic experiments to determine the response of a panel of temperate japonica accessions to mild salinity stress. A mild level of stress (6 dS m^-1^) was selected because of its relevance to observed field conditions in Europe. This mild stress decreased plant growth without inducing lethality, consistent with the results of Ahmadi et al. [30]. The results showed the expected range of variation among the checks; the well-known tolerant lines had the lowest Na/K ratios and the susceptible checks had the highest ratios. The diversity of the panel was evenly distributed between the extreme checks, indicating that some European accessions carried alleles of salinity tolerance that were efficient in situations of mild stress. Although only a mild stress and a single late time point were used, this ranking is useful information for breeders. As shown by Campbell et al. [31] and Al-Tamimi et al. [33], high-throughput image-based phenomics platforms enabling longitudinal assessment might offer a more comprehensive view of the evolution of the response of rice to salt stress over time in further experiments.

The GWAS enabled the detection of a set of distinct loci significantly associated with either salinity response indices or Na^+^ or K^+^ mass fractions. Based on our experience in comparing different GWAS approaches in previous datasets, GWASs appear sensitive to small variations in the model (e.g., differences in the number of PCA axes to control the population structure) or in the proposed analysis method for improving the computation speed (e.g., the exact method versus a method that does not involve re-estimating variances for each marker). Small methodological changes translate into different significant associations, although certain associations are generally common between analyses, as observed in maize [62]. The best compromise between computational speed, statistical power and the number of false positives is often challenging to determine for data that are not simulated. Therefore, to best explore the present dataset, we used two different approaches to GWAS analyses, a classical mixed model controlling population structure and kinship [63], and an interaction model advocated by Al-Tamimi et al. [33] for situations, such as in the present study, in which two treatments (stress and control) are compared. For the classical method, which is much less computationally intensive than the interaction method, we attempted a sub-sampling method to explore the robustness of the associations as performed by Tian et al. [55] on maize and Lafarge et al. [56] on rice. Sub-sampling was preferred over bootstrapping because of the existence of a panel structure and the simplicity of file preparation, as a given accession was not repeated several times. Sub-sampling was used to assess the effect of minor variations in MAF in the different samples and, to a lesser extent, examine the effect of population structure. Subsampling enabled us to discard associations that were detected in only in a few samples and occasionally detected in a single sample and had a high probability of being artifacts. Although sub-sampling is a satisfactory method to test the robustness of the detected associations, the time necessary for such computations and subsequent exploitation of the results did not allow the use of more than 100 sub-samples. With the development of appropriate tools, a higher number of samples (500 to 1000) might become manageable, leading to even stronger confidence in the sub-sampling outcome.

We compared the results of the classical method to those of the interaction method that was designed to manage split-plot experiments. The interaction method identified associations with much lower P values than the classical method and a higher number of markers generally supported each peak, as also observed by Al- Tamini et al. [33]. However, although the interaction model involved the control of population structure and kinship using the same matrices as the classical model, the control of false positives was not as good with the interaction method as that observed with the classical method, indicating that part of the increased power is likely obtained at the cost of an inflated rate of false discovery. In addition, the QTLs identified by the interaction method were generally different from those identified using the classical method. Only three QTLs were in common, and these QTLs were not having the lowest P values. This lack of consistency across methods may be partly due to the architecture of the traits involving a large number of QTLs with small effects, which are borderline in terms of significance. Bian et al. [62] suggested this explanation for the poor correspondence among GWAS results in maize. The lack of consistency can also result from a lack of power in the present study design, although the panel size (230 individuals) is reasonably large for plants and the phenotypic variation within the panel is extensive.

Variations in the associations detected by different methods in the discovery phase imply a need to validate the detected associations, e.g., by replicating experiments with independent panels. However, in addition to the unavoidable GxE interactions in replicating any phenotypic experiment, such replications with independent samples might be particularly difficult in a GWAS context because of the differences in structure, LD, MAF values and marker density between panels [64–65]. These differences can explain why it is challenging to obtain similar results in different GWAS. To strengthen the original evidence, other genetic methods based on gene-based haplotype analyses followed by genetic transformation [66] or on the development of bi-parental mapping populations from panel accessions with complementary haplotypes at significant clusters of markers as proposed by Phung et al. [67] might be better validation choices. However, because of the time and effort that these approaches require, they should be attempted only for the most promising cases. Functional genomic studies, notably gene expression studies, have also been used to provide evidence corroborating or invalidating the results of GWASs [68–69].

Some of the identified associations correspond to or are close to genes involved in salinity tolerance in rice (Table 7). More genes were involved in the response to ionic stress rather than osmotic stress, likely because the measured traits involve only late plant responses. Two genes (*OsCBL7* and *OsCBL8*) belonged to the ionic stress signaling pathway involving calcium. The signaling of ionic stress acts through the Ca^2+^/phospholipase C, salt overly sensitive and calmodulin pathways [34,70]. Genes related to signaling and the signal transduction of the same families were also detected by Al-Tamimi et al. [33] as an early stress response in a GWAS conducted on a panel with different genetic backgrounds (aus and indica). Salt stress induces cytosolic Ca^2+^ spiking, which in turn activates Ca^2+^ binding proteins, such as calmodulins (e.g., *OsCPK1* in Table 7). Two other kinases (e.g., *OsSAPK1* and *Os07g44330*) could also play a role in ionic stress responses.

Seven genes in Table 7 are ion transporters or transcription factors (TFs) that directly activate such genes. *HKT1;2* on chromosome 4 (a pseudogene in Nipponbare [71]), is a Na^+^/K^+^ symporter belonging to the *HKT* transporter family; the members of this family protect leaves from Na^+^ accumulation by removing Na^+^ from the xylem sap [35]. Several genes were vacuolar antiporter exchange proteins, such as high-capacity cation/H^+^ exchangers (*OsCAX1* and *OsCAX2*), a Mg^2+^/H^+^ exchanger (*OsMXH2*) or a high-affinity Ca^2+^-ATPase (*OsACA6*), that play a role in intracellular Ca^2+^-mediated sodium ion homeostasis [72–73]. These genes indicate a preferential mechanism of salt tolerance through Ca^2+^ selective accumulation in a temperate background. Zeng et al. [74] demonstrated that Na^+^-Ca^2+^ selectivity was highly correlated with yield under stress and suggested using this parameter as a selection criterion in screening. It may be worthwhile to quantify the Ca mass fraction in future studies involving temperate accessions. *OsKAT1* is a highly selective inward-rectifying K^+^ channel protein of the Shaker family, which also plays a role in cellular homeostasis [75]. The *OsKAT1* gene was present in a highly significant cluster of SNPs detected in another GWAS conducted in a temperate japonica sub-panel [31]. One gene, *OsPCF2*, is a TF that binds to the promoter of *OsNHX1*, which is itself a vacuolar K^+^-Na^+^/H^+^ antiporter that plays role in Na^+^ compartmentalization into vacuoles [70].

Four genes (*OsNAC1*, *OsNTL6* (or *ONAC070*), *OsNAC3*, and *OsNAC*5) belong to the large family of NAC (NAM, ATAF and CUC) or NAC-like TFs. Most of these genes are induced by salt stress and regulate salt-responsive genes through an ABA-dependent manner [76]. The role of *OsNTL6* and other membrane-bound TFs has been clarified [77]. Environmental changes trigger the release of the TF from the membrane to which it is anchored and, after translocating to the nucleus, this TF regulates the expression of genes involved in stress signaling and stress responses.

At least one gene is involved in the scavenging of oxygen reactive species (*OsRIP18* with superoxide dismutase activity). *OsLEA5* is a late embryogenesis abundant protein. The *LEA* genes accumulate during water stress, playing a protective role in cellular structures [17]. Two genes belong to the *BURP* family. Although *BURP* genes respond to salt stresses, their precise function is unknown [13]. *GF14E*, *OsABF2*, and *OsHsfB2b*, which are isolated members of other families, either regulate or are regulated by salt stress but there is too little information to formulate hypotheses on the precise role of these genes. Among these genes, *OsABF2* was found near a QTL for survival days of seedlings in another GWAS with a japonica panel [12].

Although the list in S2 Table includes more than 300 genes, this list is certainly not exhaustive. Therefore, we also examined genes underlying the significant associations/intervals using keywords (S4 Table). Additional potentially interesting genes were observed, but functional evidence of their involvement in salinity response is lacking. Among the transporters, *Os06g36590* (a monovalent cation/proton antiporter-2 family), *Os03g01330* (a sodium/potassium/calcium exchanger-1), *Os07g32530* (a potassium transporter) and *Os01g50860* (a chloride transporter), may be relevant. Several genes with antioxidant capacities were identified, and these genes could play a role in the protection of cellular components [13]. Several protein phosphatases, including a large cluster of serine/threonine phosphatases 2C were on the list. Protein phosphatases constitute a large family in rice, and are known to reverse the activity of protein kinases and play a functional role in ABA-mediated signaling pathways and stress responses [78]. Moreover, more than 70 receptor kinases and 80 other kinases were also on the list. Three aquaporins, *OsNIP14;1*, *OsPIP2;7* and *OsPIP1;3*, were recorded. Aquaporins mediate water transport across cellular membranes and play an important role in maintaining water homeostasis during salinity/osmotic stress [17]. Three *LEA* proteins were found in addition to *OsLEA5* (see above).

The search for candidate genes was fruitful but should not be overvalued. The first limitation of this approach is that, in this panel, full LD (r^2^ = 1.0) is often observed between remote points on the chromosomes (e.g., q02_2 or q09_01); therefore, the number of genes underlying these intervals is large, which increases the chances of finding candidates among the salt tolerance genes. Second, regarding the larger list of genes (S4 Table), although some of the candidate genes may be interesting, the functions attributed to these genes are often sufficiently vague that a plausible ex-post rationalization for their association with a trait is generally likely, which is particularly true for TFs and kinases because of their high frequency in the rice genome and large range of putative functions.

The Saltol QTL and the HKT1;5 gene that it carries affect the leaf Na^+^/K^+^ ratio at the seedling stage while they are not among the segments/genes identified as significant in the present panel. The use of the 3,000 genomes project and the data from Platten et al. [14] enabled us to show that it is likely that none or only a few of the temperate accessions carry a favorable allele at the QTL. Since the panel accessions show a large range of salinity tolerance, it is therefore likely that different mechanisms/genes were selected among the temperate accessions. This result also indicates that some progress in the salinity tolerance of the temperate accessions might be obtained by introgressing Saltol into the temperate background, as attempted in the Neurice project (http://neurice.eu/).

## Conclusion

The present study provides information on the salinity tolerance of commonly used accessions in European breeding programs. Through comparisons between GWAS models, the present study provides an idea of the variations in the associations detected by different models and highlights the interest of re-sampling methods in this context. These results emphasize the need to validate putative QTLs and candidate genes by other approaches. However, because of the importance of the associations linked to calcium-mediated ion homeostasis-related genes, the present study also gives interesting clues on which pathways are a priority to explore to obtain a better understanding of the mechanisms of salinity tolerance of temperate rice.

## Supporting information

S1 Table List of accessions in the panel and their phenotypic data registered in the hydroponic experiment. (XLSX)

S2 Table Comparison of models with kinship alone (MLM1) and kinship and structure (MLM2) using the AIC. (DOCX)

S3 Table List of genes involved in salinity tolerance in rice. (XLSX)

S4 Table Matrices of LD (r^2^) among significant markers using classical/interaction methods. (XLSX)

S5 Table List of all genes with annotated functions surrounding the significant markers/intervals (100 kb). (XLSX)

S1 Fig Phenotypic correlation among indices of stress response. iTIL: relative number of tillers; iLL: relative maximum leaf length; iRL: relative maximum root length, iROOT: relative root dry weight; iSHOOT: relative shoot dry weight; iR/S: relative root-to-shoot ratio; iLA: relative leaf area; and iSLA: relative specific leaf area.
(PDF)

S2 Fig PCA on the indices of stress response and ion parameters. (PDF)

S3 Fig QQ plots for the two tested models. A. Model with kinship alone. B. Model with structure and kinship.
(PPTX)

S4 Fig Manhattan plots for the 8 growth traits analyzed with the conventional method. iTIL: relative number of tillers; iLL: relative maximum leaf length; iRL: relative maximum root length, iROOT: relative root dry weight; iSHOOT: relative shoot dry weight; iR/S: relative root-to-shoot ratio; iLA: relative leaf area; and iSLA: relative specific leaf area.
(PPTX)

S5 Fig NJ tree based on the sequences of the *HKT1;5* gene. (PDF)
