# Supplementary material for: Tolerance to mild salinity stress in japonica rice: A genome-wide association mapping study highlights calcium signaling and metabolism genes
Source: PLoS One. 2018 Jan 17;13(1):e0190964. doi: 10.1371/journal.pone.0190964 (PMC5771603; doi:10.1371/journal.pone.0190964)
Supplement: S2 Table — (DOCX) [file pone.0190964.s002.docx]

**S2 Table. Comparison of models with kinship alone (MLM1) and kinship and structure (MLM2) using an Akaike Information Criterion (AIC).**

|  | MLM1 | | MLM2 | |
| --- | --- | --- | --- | --- |
| Trait | -2Ln(L) | AIC | -2Ln(L) | AIC |
| iTIL | 2031.07 | 2037.07 | 2007.39 | 2017.39 |
| iLL | 1520.09 | 1526.09 | 1506.55 | 1516.55 |
| iRL | 1834.04 | 1840.04 | 1812.92 | 1822.92 |
| iROOT | 2007.00 | 2013.00 | 1987.93 | 1997.93 |
| iSHOOT | 1918.88 | 1924.88 | 1899.81 | 1909.81 |
| iLA | 1671.26 | 1677.26 | 1653.16 | 1663.16 |
| iSLA | 1667.13 | 1673.13 | 1645.98 | 1655.98 |
| iR_S | 1805.64 | 1811.64 | 1782.26 | 1792.26 |
| Na | -187.18 | -181.18 | -191.19 | -181.19 |
| K | 58.93 | 64.93 | 52.76 | 62.76 |
| Na_K | -394.38 | -388.38 | -398.85 | -388.85 |

The AIC was computed as -2 ln(L) + 2k where ln is the natural logarithm, L is the maximized value of the likelihood function for the estimate model and k is the number of parameters fitted in the model [54]. The model with the smallest AIC was selected.

iTIL: relative number of tillers; iLL: relative maximum leaf length; iRL: relative maximum root length, iROOT: relative root biomass; iSHOOT: relative shoot biomass; iLA: relative leaf area; iSLA: relative specific leaf area; iR_S: relative root-to-shoot ratio; Na: sodium mass fraction in the leaves; K: potassium mass fraction in the leaves; Na_K: Sodium-to potassium ratio in the leaves.
