# Supplementary figures and images for: Tolerance to mild salinity stress in japonica rice: A genome-wide association mapping study highlights calcium signaling and metabolism genes
Source: PLoS One. 2018 Jan 17;13(1):e0190964. doi: 10.1371/journal.pone.0190964 (PMC5771603; doi:10.1371/journal.pone.0190964)

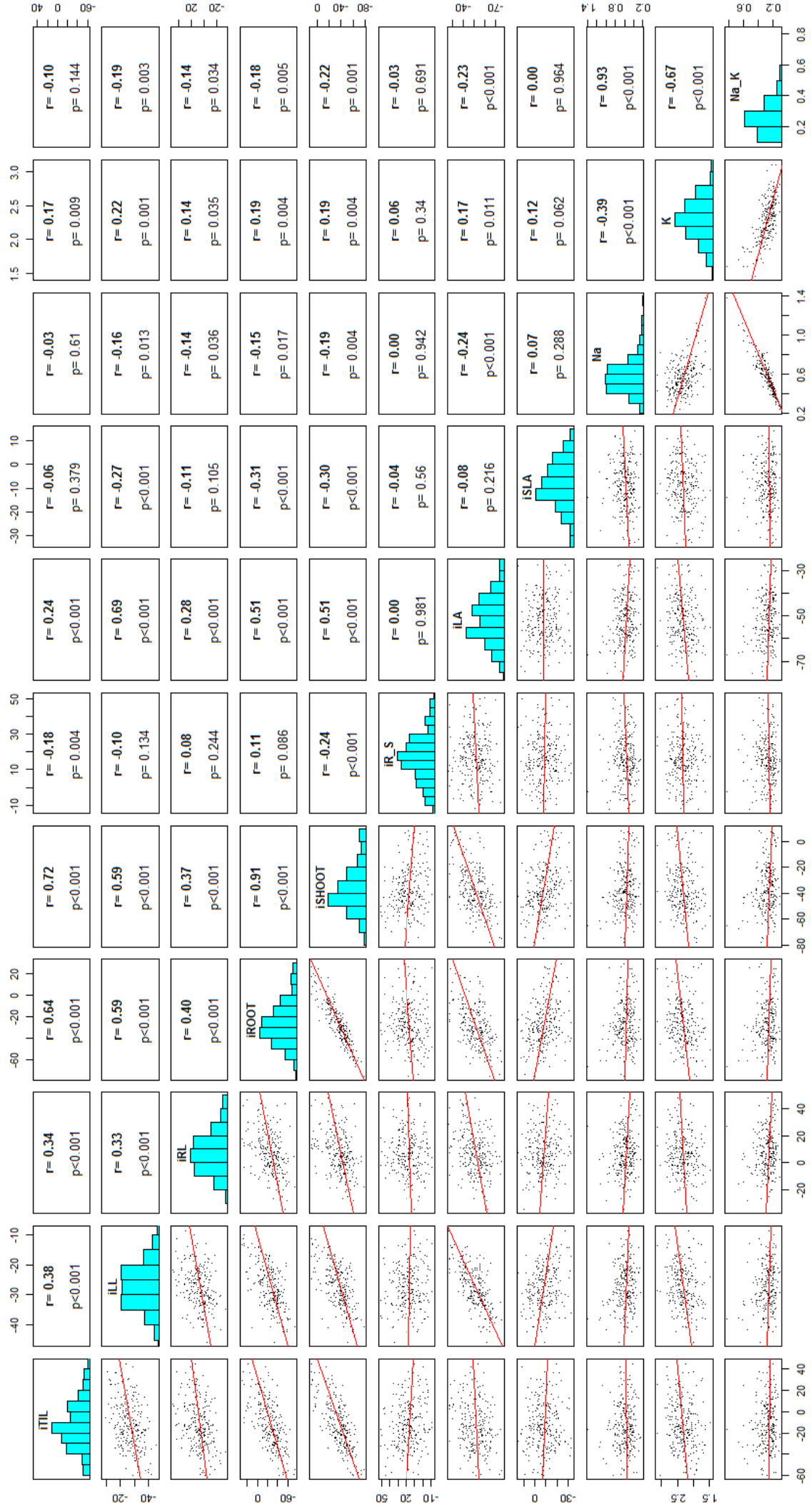

Supplement: S1 Fig — iTIL: relative number of tillers; iLL: relative maximum leaf length; iRL: relative maximum root length, iROOT: relative root dry weight; iSHOOT: relative shoot dry weight; iR/S: relative root-to-shoot ratio; iLA: relative leaf area; and iSLA: relative specific leaf area. (PDF) [file pone.0190964.s006.pdf]

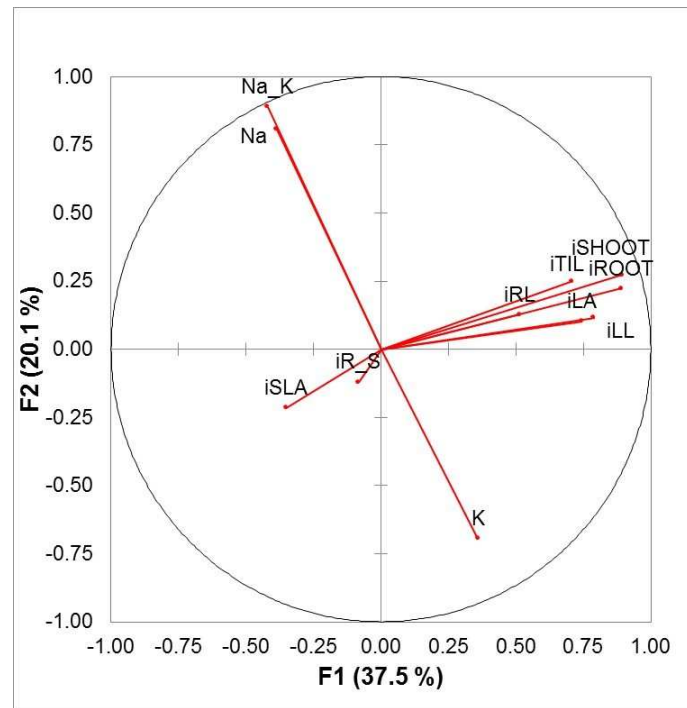

Supplement: S2 Fig — (PDF) [file pone.0190964.s007.pdf]

## Slide 1
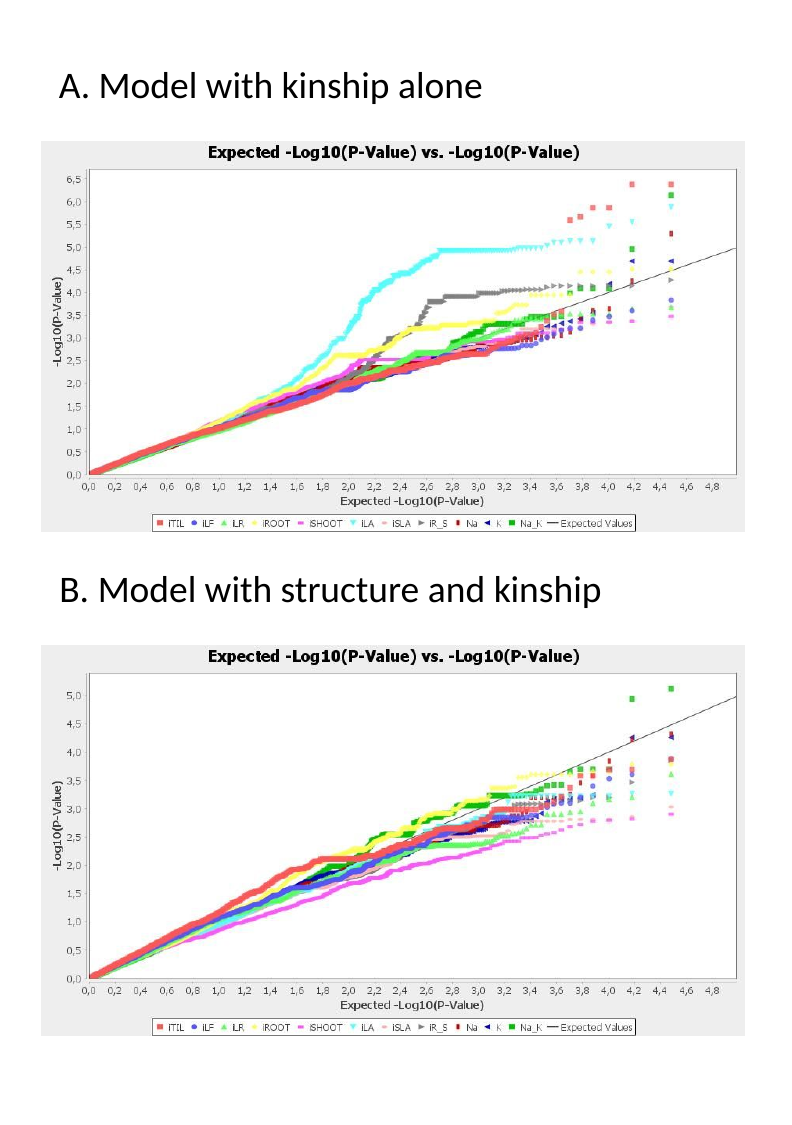

A. Model with kinship alone
B. Model with structure and kinship

Supplement: S3 Fig — A. Model with kinship alone. B. Model with structure and kinship. (PPTX) [file pone.0190964.s008.pptx]
