# Supplementary material for: Tolerance to mild salinity stress in japonica rice: A genome-wide association mapping study highlights calcium signaling and metabolism genes
Source: PLoS One. 2018 Jan 17;13(1):e0190964. doi: 10.1371/journal.pone.0190964 (PMC5771603; doi:10.1371/journal.pone.0190964)
Supplement: S4 Fig — iTIL: relative number of tillers; iLL: relative maximum leaf length; iRL: relative maximum root length, iROOT: relative root dry weight; iSHOOT: relative shoot dry weight; iR/S: relative root-to-shoot ratio; iLA: relative leaf area; and iSLA: relative specific leaf area. (PPTX) [file pone.0190964.s009.pptx]

## Slide 1
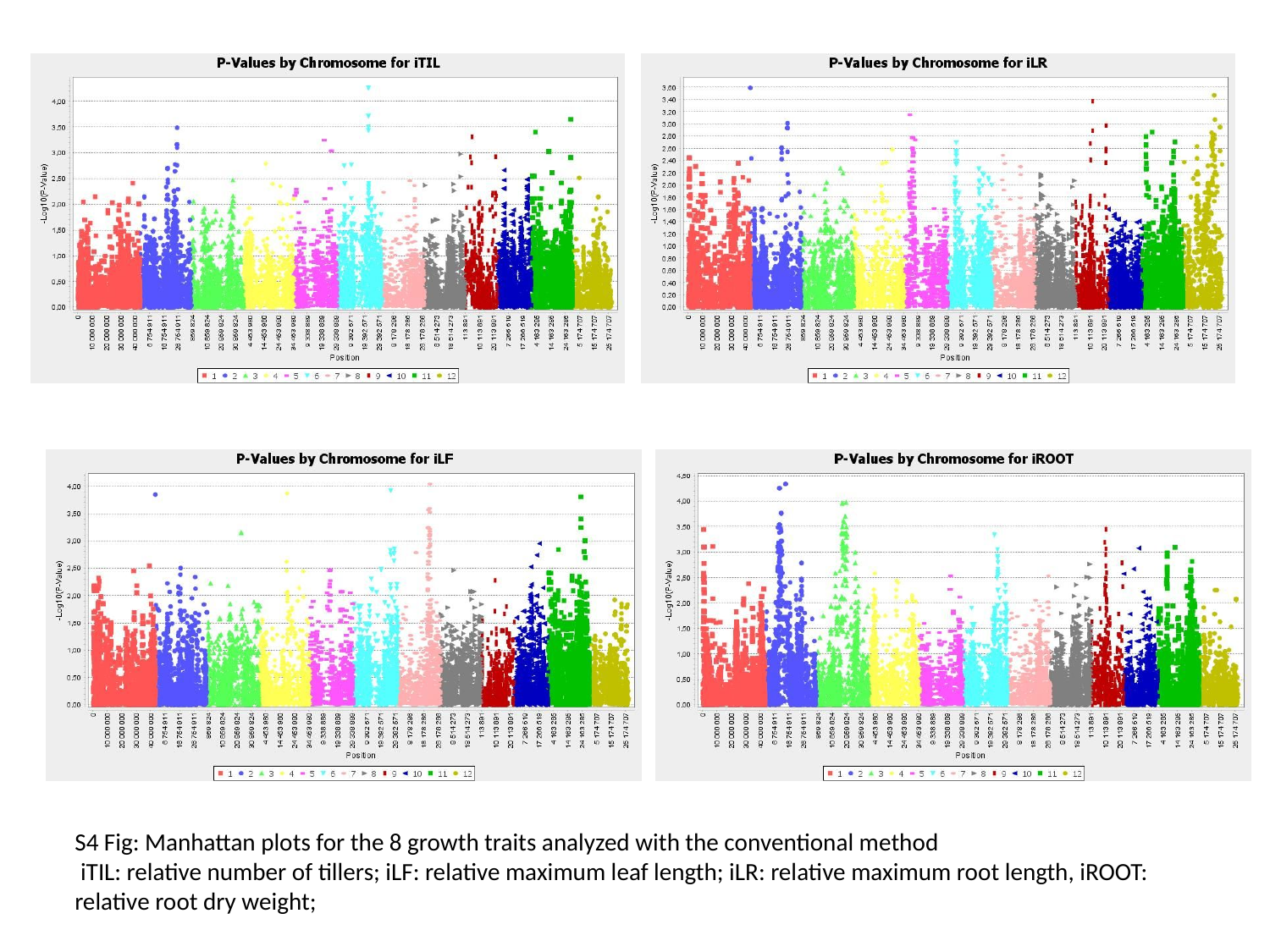

S4 Fig: Manhattan plots for the 8 growth traits analyzed with the conventional method
 iTIL: relative number of tillers; iLF: relative maximum leaf length; iLR: relative maximum root length, iROOT: relative root dry weight;

## Slide 2
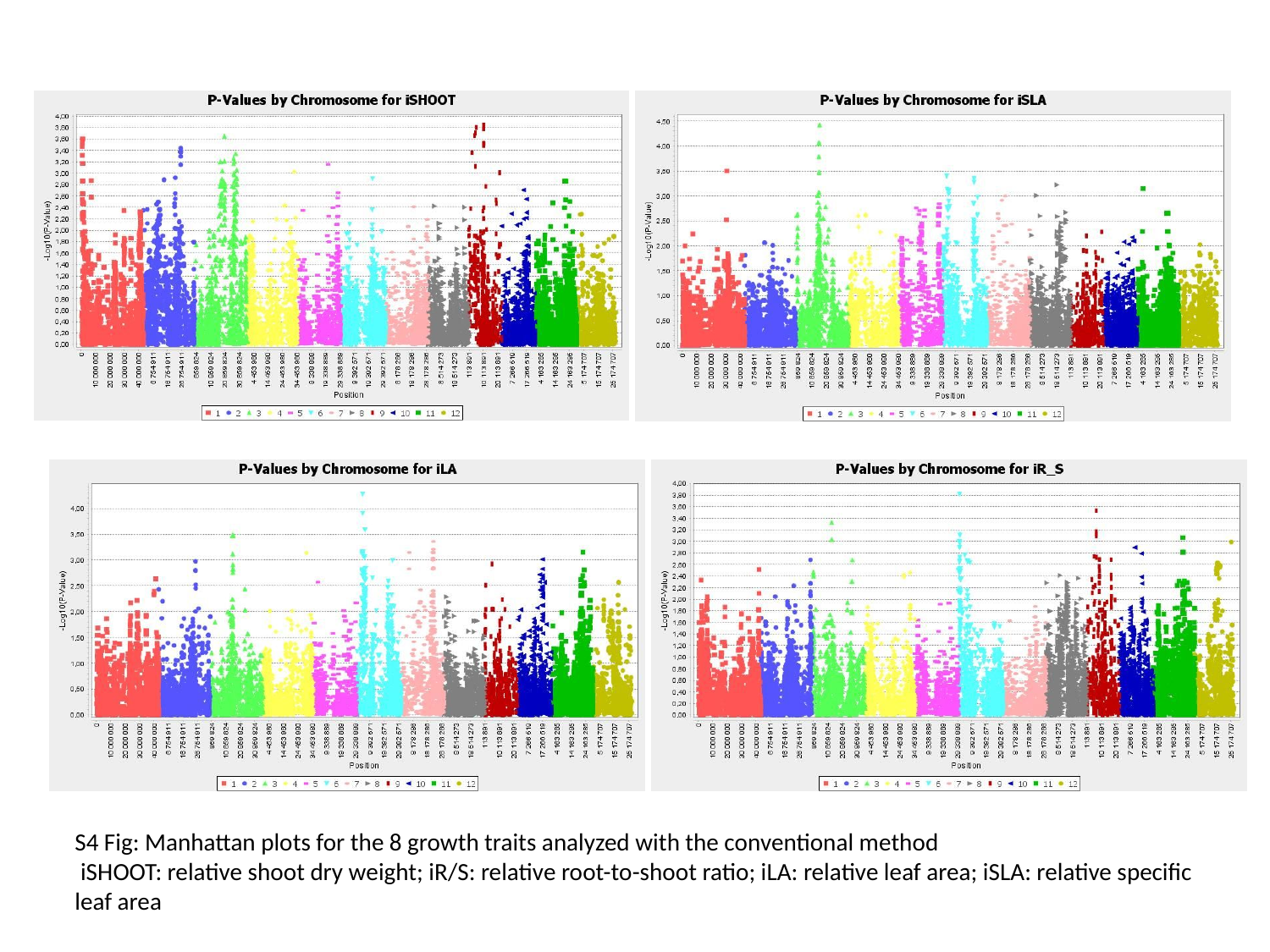

S4 Fig: Manhattan plots for the 8 growth traits analyzed with the conventional method
 iSHOOT: relative shoot dry weight; iR/S: relative root-to-shoot ratio; iLA: relative leaf area; iSLA: relative specific leaf area
