# Supplementary material for: Tolerance to mild salinity stress in japonica rice: A genome-wide association mapping study highlights calcium signaling and metabolism genes
Source: PLoS One. 2018 Jan 17;13(1):e0190964. doi: 10.1371/journal.pone.0190964 (PMC5771603; doi:10.1371/journal.pone.0190964)
Supplement: S5 Fig — (PDF) [file pone.0190964.s010.pdf]

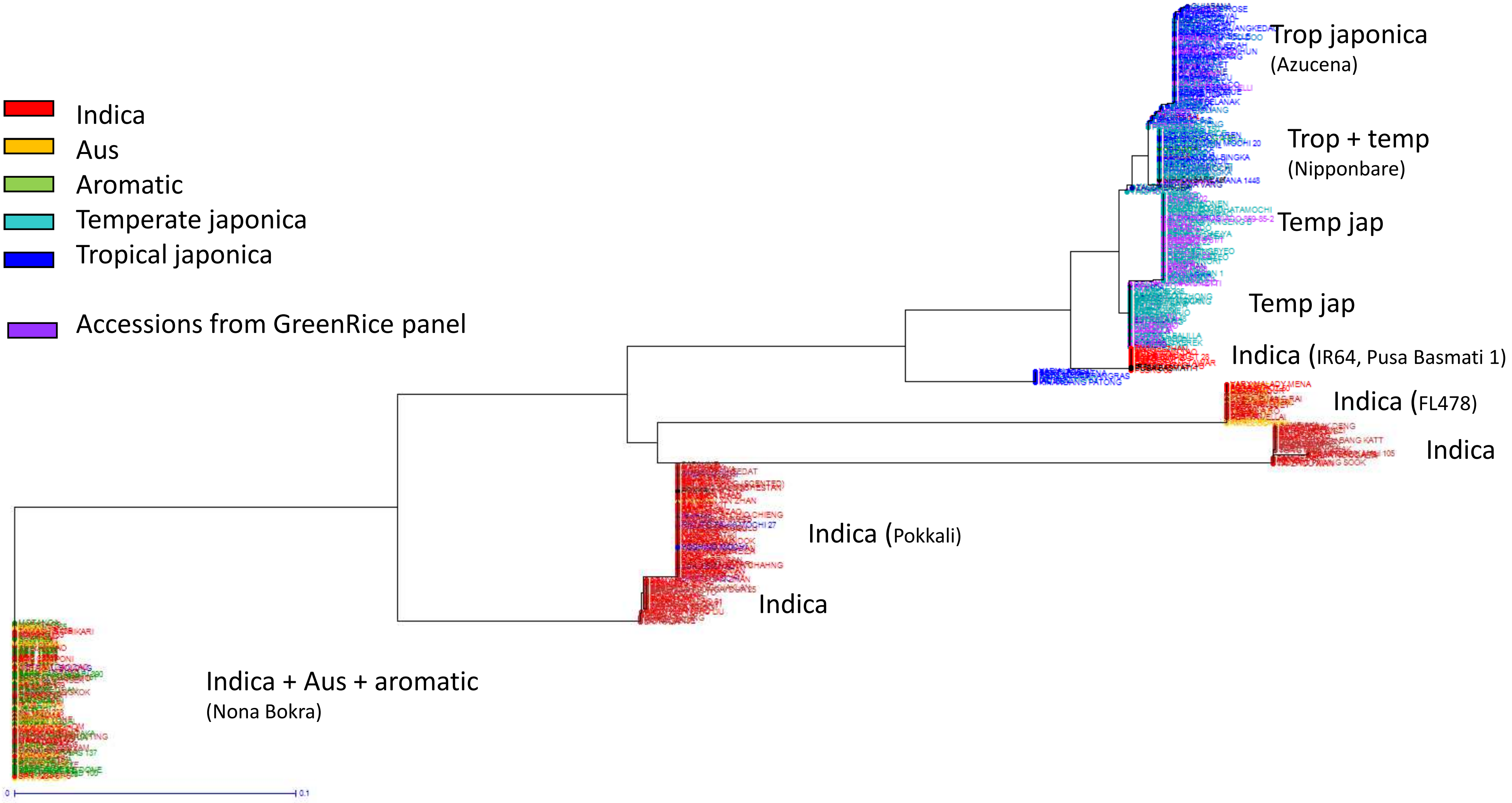
Indica
Aus
Aromatic
Temperate japonica
Tropical japonica
Accessions from GreenRice panel
Trop japonica
(Azucena)
Trop + temp
(Nipponbare)
Temp jap
Temp jap
Indica (IR64, Pusa Basmati 1)
Indica (FL478)
Indica
Indica (Pokkali)
Indica
Indica + Aus + aromatic
(Nona Bokra)
